# Twenty patients including 7 probands with autosomal dominant cutis laxa confirm clinical and molecular homogeneity

**DOI:** 10.1186/1750-1172-8-36

**Published:** 2013-02-25

**Authors:** Smail Hadj-Rabia, Bert L Callewaert, Emmanuelle Bourrat, Marlies Kempers, Astrid S Plomp, Valerie Layet, Deborah Bartholdi, Marjolijn Renard, Julie De Backer, Fransiska Malfait, Olivier M Vanakker, Paul J Coucke, Anne M De Paepe, Christine Bodemer

**Affiliations:** 1 Service de Dermatologie – Centre de référence national des Maladies Génétiques à Expression Cutanée (MAGEC), INSERM U781, Hôpital Necker - Enfants Malades, Université Paris V-Descartes, 149, rue de Sèvres 75743 Paris Cedex 15, Paris, France; 2 Center for Medical Genetics, Ghent University Hospital, De Pintelaan 185, B-9000, Ghent, Belgium; 3 Service de Dermatologie, MAGEC Hôpital Saint-Louis, Paris, France; 4 University Medical Center St. Radboud, Nijmegen, the Netherlands; 5 Academic Medical Center, Amsterdam, the Netherlands; 6 Groupe Hospitalier Du Havre – Department of Medical Genetics, Le Havre, France; 7 Institute of Medical Genetics, University of Zürich, Zürich, Switzerland

**Keywords:** Elastin, *ELN*, Autosomal dominant cutis laxa, Genotype, Phenotype

## Abstract

**Background:**

Elastin gene mutations have been associated with a variety of phenotypes. Autosomal dominant cutis laxa (ADCL) is a rare disorder that presents with lax skin, typical facial characteristics, inguinal hernias, aortic root dilatation and pulmonary emphysema. In most patients, frameshift mutations are found in the 3’ region of the elastin gene (exons 30-34) which result in a C-terminally extended protein, though exceptions have been reported.

**Methods:**

We clinically and molecularly characterized the thus far largest cohort of ADCL patients, consisting of 19 patients from six families and one sporadic patient.

**Results:**

Molecular analysis showed C-terminal frameshift mutations in exon 30, 32, and 34 of the elastin gene and identified a mutational hotspot in exon 32 (c.2262delA). This cohort confirms the previously reported clinical constellation of skin laxity (100%), inguinal hernias (51%), aortic root dilatation (55%) and emphysema (37%).

**Conclusion:**

ADCL is a clinically and molecularly homogeneous disorder, but intra- and interfamilial variability in the severity of organ involvement needs to be taken into account. Regular cardiovascular and pulmonary evaluations are imperative in the clinical follow-up of these patients.

## Background

Elastin gene (*ELN*) mutations have been associated with various skin, cardiovascular and pulmonary phenotypes. Microdeletions of the region on 7q11, encompassing the *ELN* gene, result in Williams-Beuren syndrome (WBS, OMIM #194050) [1]. This recognizable entity combines mental retardation with a happy demeanor, typical facial gestalt, a soft, slightly elastic and doughy skin, hypercalcaemia, and arterial stenoses, mainly of the supravalvular aorta. A related disorder is the supravalvular aortic stenosis (SVAS, OMIM #185500) phenotype. These patients present without the mental and dysmorphic features encountered in the WBS, but do show arterial stenoses. SVAS is caused by missense and premature truncation mutations that are dispersed throughout the *ELN* gene [2].

In contrast, frameshift mutations at the 3’ terminus that result in a C-terminally elongated and secreted elastin protein [3-8] have been identified in autosomal dominant cutis laxa (ADCL, OMIM #123700). This disorder comprises generalized skin redundancy, pulmonary emphysema and aortic root dilatation (ARD).

Different mechanisms underlying the perturbations subsequent to elastin mutations have been identified. In general, reduced elastin secretion and deposition, resulting in increased vascular smooth muscle cell proliferation and a higher number of smaller elastic lamellae, induces a phenotype dominated by arterial stenoses [9], while aberrant elastin protein production infers to elastic fiber fragmentation with manifest cutis laxa, arterial dilatation and/or emphysema [3].

To date, frameshift mutations in the 3’UTR of the elastin gene have been identified in 11 probands with ADCL, but it remains a matter of debate whether other molecular mechanisms could be involved. Indeed, two reports indicated a different molecular basis including a heterozygous mutation in exon 25 of the elastin gene [10], and a tandem duplication in the fibulin-5 gene [11], respectively. Moreover, in the absence of large patient cohorts, the ADCL phenotype is still insufficiently characterized and not always easy to discern from related forms of cutis laxa. Furthermore, the identification of an *ELN* variant in exon 34 (c.2318G>A; p.(G773D)) complicated genotype-phenotype correlations in the *ELN* gene. This variant was associated with pulmonary emphysema [12] and, in conjunction with a p.G202R alteration in the fibulin-5 protein, with acquired cutis laxa following a parasitic infection [13]. Finally, a case report mentions causal involvement of the *ELN* gene in a recessive form of cutis laxa due to a p.(P211S) alteration [14].

We report on six novel ADCL families, comprising 19 patients, and one sporadic patient, with a review of the literature. Our data favor homogeneity both at the clinical and molecular level.

## Methods

### Clinical data

Patients were evaluated by an experienced clinical geneticist or dermatologist. Written informed consent was obtained from each patient and family member before sample collection. Specific consents were obtained for publication of the clinical pictures. This study was approved by the medical ethical committee of Inserm (France) and of the Ghent University Hospital and the Declaration of Helsinki Principles were respected.

### Mutation screening

Genomic DNA was extracted from peripheral blood leukocytes at Genethon’s DNA Bank using standard procedures, followed by touchdown PCR amplification and sequencing of exons 28 through 34 of the *ELN* gene as previously described [3]. Sequences were compared to the wild-type sequence as submitted to Ensembl Accession number ENST00000358929. Nucleotides were numbered starting from the first base of the initiation codon (ATG) of the cDNA reference sequence. Amino acid residues are numbered from the first methionine residue of the reference sequence. Segregation was verified in all families and mutations were absent in a panel of 100 Caucasian controls.

## Results

### Clinical data

**Figure 1:**
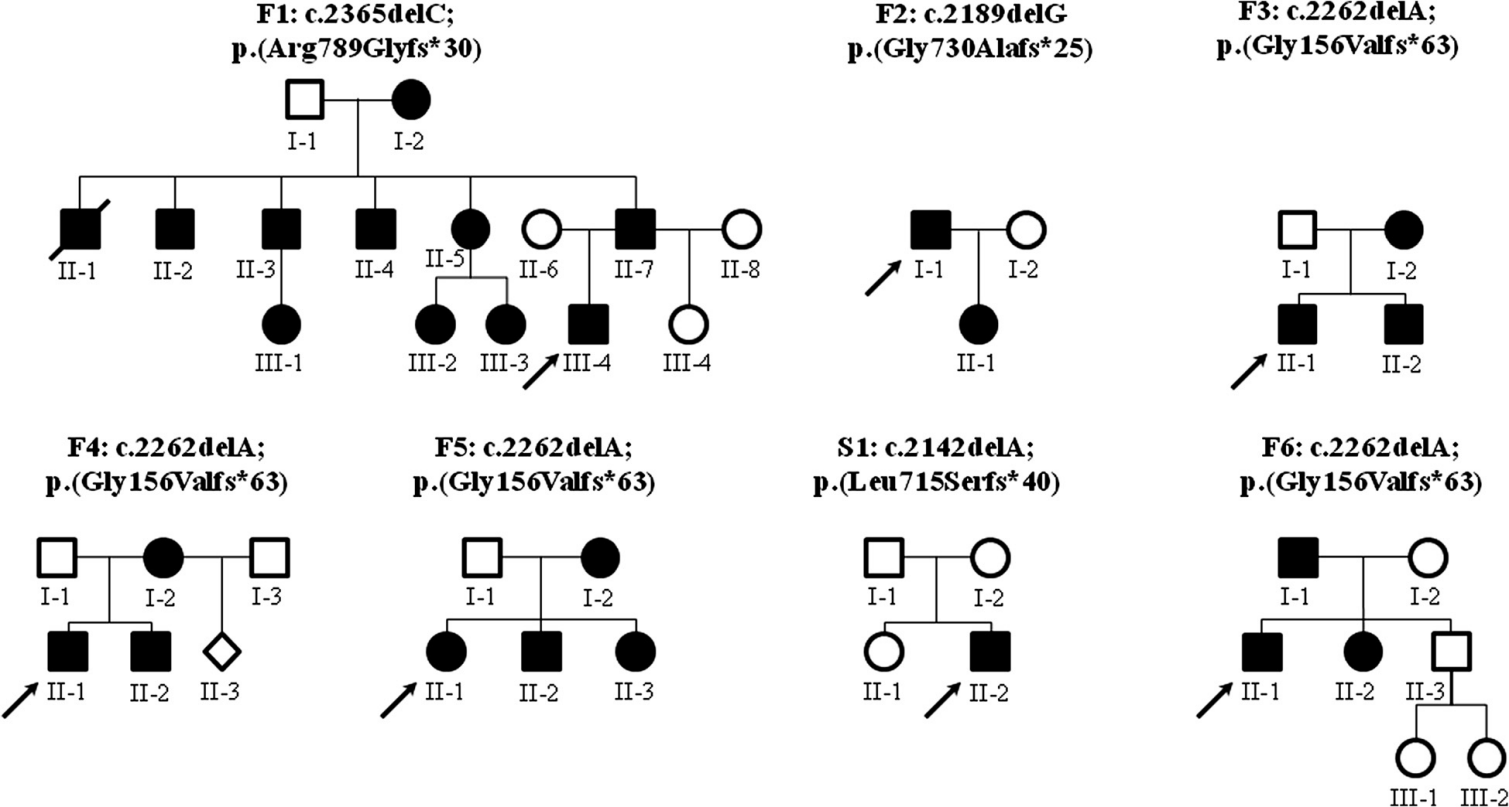
**Pedigrees of the families described in this study.** Round symbol, female; square, male; filled symbol, affected; open symbol, normal; slash line, deceased.

We present six families and one sporadic patient (S1:II-2) with ADCL. Pedigrees of the families are presented in Figure 1. Family 1 originates from the Netherlands, Family F2 from Switzerland, and all others from France. Twenty of the 27 patients were available for clinical evaluation (Table 1). Male to female ratio was 15/12. Curiously, in 20 out of 22 meioses the phenotype was inherited (*Χ*^2^=8.84; p-value=0.003). Ages at last clinical evaluation ranged from 1 to 84 years, with 11 patients being over 21 years old.

**Table 1:** Clinical details of the patients available for clinical evaluation

**Patient**	**Sex, Age (y)**	**Skin**							**Inguinal hernia**	**Cardiovascular**	**Pulmonary emphysema**	**Other**
		**Aspect**	**Topography**						**Left (L)/Right (R)/Bilateral (B) (times surgery)**			
			**Trunk**	**Axillar region**	**Inguina region**	**Limbs**	**Neck**	**Face**				
F1:I-1	F, 84	Redundant	+	+	+				L (1)	-	+	Bilateral ptosis, sagging breasts, pelvic prolapse
F1:II-2	M, 59	Smooth, sagging, loose		+	+		+	+	B (10).	-	+	Pliant ears
F1:II-3	M, 57	Smooth, redundant sagging	+	+				+	B (12)	TVS ARD	+	Nycturia, decreased fertility
F1:II-4	M, 53	Sagging loose, sagging, redundant		+	+	+	+	+	B (15)	ARD (85mm – surgery), AR, MR	+	Umbilical hernia
												Inguinal hernia surgery (13 times)
F1:II-5	F, 51	Sagging loose, sagging, redundant	+	+	+		+	+	-	-	-	
F1:II-7	M, 46	Loose, sagging	+				+	+	-	-	-	Gastroesophageal reflux disease, strabismus
F1:III-1	F, 23	Sagging, loose, redundant	+	+	+		+		B (2)	-	-	Sagging breasts
F1:III-4	M, 22	Sagging	+					+	R (2)	ARD	-	Gastroesophageal reflux disease, coarse face
F2:I-1	M, 48	Redundant		+	+				-	-	+	Hoarse voice, corrective skin surgery
F2:II-1	F, 21	Redundant		+	+				B (0)	MVP	+	Hoarse voice, corrective skin surgery (3 times)
F3:I-2	F, 31	Excessive, sagging	+	+	+	+	+	+	-	ARD	-	
F3:II-1	M, 7	Excessive, sagging					+	+	-	ARD	-	Hoarse voice
F3:II-2	M, 4	Excessive, sagging	+	+	+	+	+	+	-	Pulmonary artery dilatation	Asthma	Atopic dermatitis, hoarse voice
F4:II-1	M, 16	Redundant	+	+	+			+	-	ARD		Coarse face
F4:II-2	M, 14	-						+	-	Dilated aortic arch (27mm)		Coarse face
F5:II-1	F, 14	Excessive, sagging	+	+	+	+	+	+	-	-	-	Labiae major coalescence
F5:II-2	M, 10	Excessive, sagging	+	+	+	+	+	+	-	AR	-	Testis ectopia
F5:II-3	F,7	Excessive, sagging	+	+	+	+	+	+	-	AR, Borderline ARD (23mm)	-	
S1:II-2	M, 1	Excessive, sagging,	+	+	+			+	-	ARD		
F6:II-1	M, 39	Redundant	+	+				+	-	AR, global increase of arterial diameter	-	

*M*, male; *F*, female; *y*, years; *L*, left; *R*, right; *B*, bilateral; *AR*, aortic valve regurgitation; *ARD*, aortic root dilatation; *MVP*, mitral valve prolapse; *MR*, mitral valve regurgitation; *TVS*, tricuspid valve stenosis. F1 to F6: familial cases, S1: sporadic case.

**Figure 2:**
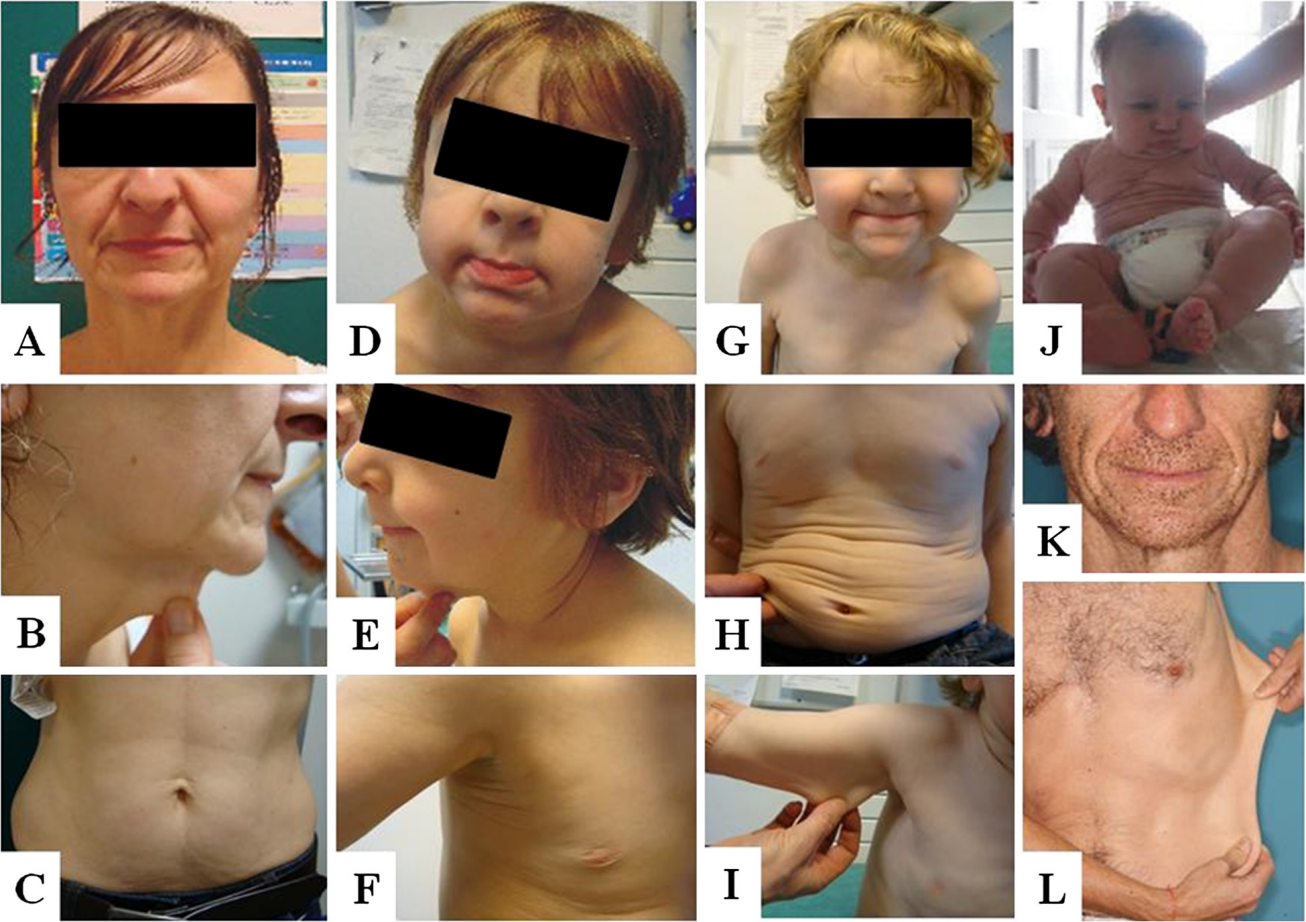
**Clinical aspects of affected patients.** Facial characteristics include large ears, a long, coarse face, blepharochalasis, ptosis, a beaked nose and a large philtrum. Note the variability of skin phenotype at different ages. (**A-C**) Typical facial characteristics, slightly hyperextensible skin, mild rimpling and sagging of the abdominal skin in patient F3:I-2. (**D-F**) Typical facial characteristics, slightly hyperextensible skin, and small redundant skin folds of the axillar folds in patient F3:II-1. (**G-I**) Typical facial characteristics, manifest loose, redundant and sagging skin folds of the abdominal skin and slightly hyperextensible skin of the limbs in patient F3:II-2. (**J**) Patient S1:II-2 at the age of 1 year presenting with typical facial characteristics and generalized, loose, redundant skin folds. (**K-L**) Facial characteristics and skin hyperextensibility in patient F6:II-1.

All patients had areas of cutis laxa. A minority of patients (25%) showed skin redundancy only in the facial, neck, inguinal and/or axillary regions, while 75% of patients had extensive to generalized cutis laxa. The facial skin showed a spectrum ranging from a coarse face over an aged appearance to overt cutis laxa. Especially the lower half of the face and neck region were involved with sagging cheeks, a flabby skin around the chin and skin redundancy in the neck region. Blepharochalasis and ptosis of the upper eyelid were often present (Figure 2J). The skin of the limbs was relatively spared. Overall, the skin had a soft feeling to the touch with variable hyperextensibility (Figure 2L). Skin abnormalities tended to improve with age but were highly variable between and within families. Surgery both for aesthetic reasons [sagging breasts (F1:II-2, F1:III-1) or lax abdominal skin (F2:I-1 and F2:II-1)] and for functional reasons [ptosis (F1:II-2)] was disappointing with frequent relapse. Wound healing was reported normal, although F1:III-1 mentioned recurrent superficial erosions, wound infections and nail fragility.

Craniofacial dysmorphism was mild but recognizable and showed a long face, large pliant ears, and a long philtrum (Figure 2). A beaked nose was present in most patients (70%).

Inguinal hernias were frequent, appearing congenitally (F1:II-2, F1:II-3, F1:III-1, F2:II-1) or throughout life (F1:I-2 at age 80 and F1:III-4 at 18 years old) and often reoccurred after surgery (patient F1:II-2, F1:II-3, F1:II-3, F1:III-1). Patient F1:II-4 also had an umbilical hernia. Systemic involvement was noted in 15 out of 20 patients (75%). Chronic obstructive pulmonary disease was diagnosed in eight patients, seven of whom had emphysema and one had asthma. F1:II-2 and F1:II-1 were significant smokers and the latter died at age 55 due to end-stage respiratory failure with emphysematous lung changes. Vascular involvement included ARD in eight patients, and a dilatation of the aortic arch in one (patient F4:II-2). In addition, patient F3:II-2 had pulmonary artery dilatation and patient F6:II-1 had a global but stable increase of the diameters of the arteries, especially of the carotid arteries (diameter increase of 30%). Four patients had aortic valve regurgitation (F1:II-4; F5:II-2, F5:II-3, F6:II-1), patient F1:II-3 had tricuspid valve stenosis, and patients F1:II-4 and F6-20 had mitral valve regurgitation. Other manifestations included gastro-oesophageal reflux (F1:II-7 and F1:III-4), urogenital prolapse (F1:II-2), and a hoarse voice (F2:I-1, F2:II-1, F3:II-1, and F3:II-2).

Hypomobility of spermatozoids required *in vitro* fertilization for one patient (F1:II-3). None of the patients reported ocular problems. Growth and psychomotor development were appropriate in all patients.

### Genetic analysis

The *ELN* mutations identified in these families are reported in Table 2 and Figure 1. We found three novel mutations: p.2189delG; p.(Gly730Alafs*25) and c.2142delG; p.(Leu715Serfs*40) in exon 30; and c.2365delC; p.(Arg789Glyfs*30) in exon 34. One previously reported mutation (c.2262delA (p.Gly156Valfs*63)) in exon 32 was found in four families and may represent a mutational hotspot. All mutations are predicted to result in a C-terminal missense sequence with a read-through in the 3’ UTR. Although exon 30 mutations induce a premature truncation in exon 32, (partial) splicing of this exon will result in a C-terminally elongated missense protein as previously reported for similar mutations [3,5].

**Table 2:** ***ELN *mutations identified in this cohort**

**Patients**	**Origin**	**cDNA level**	**Protein level**
Family 1	Netherlands	c.2365delC (exon 34)	p.(Arg789GlyfsX30)
Family 2	Switzerland	c.2189delG (exon 30)	p.(Gly730AlafsX25)
Family 3	France	c.2262delA (exon32)	p.(Gly156ValfsX63)
Family 4	France	c.2262delA (exon32)	p.(Gly156ValfsX63)
Family 5	France	c.2262delA (exon32)	p.(Gly156ValfsX63)
Sporadic case	France	c.2142delG (exon 30)	p.(Leu715Serfsx40)
Family 6	France	c.2262delA (exon32)	p.(Gly156ValfsX63)

### Genotype-phenotype correlation

There are no obvious genotype-phenotype correlations when comparing patients with mutations in different exons (Table 3). The recurrent c.2262delA mutation shows large interfamilial variability. Moreover, family 1, 3 and 5 also document high intrafamilial variability, both in skin features and internal organ involvement.

**Table 3:** Genotype – phenotype correlations in ADCL

**Missense**	**F/M individuals**	**Mean age**	**FH**	**IH**	**FG**	**ARD**	**BAV**	**VA**	**Emphys**	**transmission**
**Exon 30**	**5/7**	**21.9**	**50**	**64**	**100**	**82**	**10**	**70**	**40**	**100**
**Exon 32**	**6/6**	**17.1**	**90**	**17**	**100**	**50**	**0**	**25**	**0**	**95**
**Exon 33**	**2/2**	**21**	**33**	**50**	**100**	**50**	**25**	**50**	**100**	**100**
**Exon 34**	**3/5**	**49.5**	**100**	**75**	**100**	**38**	**0**	**25**	**50**	**91**
**Chr rearr**	**3/0**	**42**	**100**	**67**	**100**	**?**	**0**	**0**	**100**	**56**
**Total**	**19/20**	**27.3**	**63**	**50**	**100**	**57**	**5**	**38**	**35**	**83**

Frequencies (%) of the clinical characteristics are given for mutations in the different exons. Chr rear, Chromosomal rearrangement as described by [7]. Mean age in years. *F*, female; *M*, male; *FH*, family history; *IH*, Inguinal hernia; *FG*, Facial gestalt; *ARD*, aortic root dilatation; *BAV*, bicuspid aortic valve; *VA*, other valve anomalies; Emphys, emphysema. References: [3-8].

Although the risk for ARD was suggested to be smaller in patients with exon 32 mutations [3], we did identify vascular involvement in this patient subgroup. However, ARD seems more prominent in association with exon 30 mutations (Table 3), but further confirmation is needed.

## Discussion

We present the largest patient cohort with ADCL thus far. The phenotype is homogeneous and comprises generalized skin involvement, inguinal and umbilical hernias, ARD and emphysema. The disorder shows full penetrance, but with variable expression. The aspect and topography of the skin lesions is highly variable but the skin changes are most pronounced on the axillar, inguinal, neck and lower facial regions. In congruence with earlier reports [5,8], skin manifestations improve with aging and the phenotype may be unrecognized in older patients. Although ADCL is often considered ‘benign’, systemic involvement, although variable, is frequently present. Both lung (present in 35% of patients) and aortic involvement (present in 57% of patients) have led to premature death [5,7]. Patient management therefore warrants close monitoring of pulmonary function and aortic root diameters. No current treatment guidelines are available for ADCL patients. Because earlier reports indicated increased TGFβ signaling in the pathogenesis [3], thresholds for aortic surgery could be based on similar parameters as in elastinopathies/TGFβ signalopathies associated with ARD (actual aortic diameter, rate of aortic growth, family history, the patient’s estimation of the risk, and pregnancy wish) [15]. Moreover, this mechanism might reveal a role for treatment with losartan, an angiotensin II type 1 receptor blocker with TGFβ antagonistic effect. Surgery for skin manifestations and hernias is disappointing and should be discouraged if done purely for aesthetic reasons.

The phenotype can be distinguished from autosomal recessive types of cutis laxa (ARCL) based on facial gestalt, the absence of skeletal, ocular, and central nervous involvement (type 2 and 3 ARCL) [16,17], and the absence of severe early onset emphysema and gastrointestinal and genitourinary diverticula (type 1a/c ARCL [18]) or arterial involvement (type 1b related cutis laxa) [19] (Table 4). The small number of patients described to date does not allow drawing straight-forward genotype-phenotype correlations, although a tendency for a milder vascular phenotype exists in exon 32 mutations. Mutations in this exon might be partially rescued by enhanced splicing of this exon [3].

**Table 4:** Clinical characteristics of ADCL and type 1 recessive cutis laxa syndromes

	**ADCL**	**ARCL type Ia**	**ARCL type 1c**	**ARCL type 1b**
	**(*ELN*)**	**(*FBLN5*)**	**(*LTBP4*)**	**(*FBLN4*)**
**Dysmorphism**	Long philtrum, beaked nose	Long philtrum, large ears, beaked nose	Long philtrum, large ears, beaked nose, sparse hair on temporal sides	
**Skin**	Loose redundant skin folds	Loose redundant skin folds	Loose redundant skin folds	Hyperextensible skin
	Coarse face			Skin redundancy
**Cardiovascular**	ARD, valvular anomalies	SVAS, PPAS	PPAS, CPAS	ARD, multiple arterial aneurysms
**Emphysema**	+	+++	+++	+/-
**Genitourinary diverticula**	+	+++	++	?
**Gastro-intestinal diverticula/tortuosity**	-	+	+++	?
**Inheritance**	AD	AR	AR	AR

*ARD*, aortic root dilatation; *SVAS*, supravalvular aortic root stenosis; *PPAS*, peripheral pulmonary artery stenosis; *CPAS*, common pulmonary artery stenosis; *AD*, autosomal dominant; *AR*, autosomal recessive. References: [3-8,18-22].

The clinical homogeneity of the phenotype corresponds to a striking molecular homogeneity with mutations found in the 3’terminus of the *ELN* gene. All mutations reside in exons 30, 32, 33, and 34 and result in a missense and elongated sequence at the C-terminus of the elastin protein [3-8]. Mutation analysis should focus firstly on these exons, although splice site mutations elsewhere in the *ELN* gene could also result in a C-terminal extension of the protein. Frameshift mutations resulting in C-terminal protein extensions have been identified in a variety of other diseases resulting in mislocalization, altered secretion, increased or decreased stability, resistance to proteases, loss-of-function, gain-of-function, and novel functions of the affected protein [23-28]. Extended proteins may also result from impaired processing of propeptides. A well-known example within the field of connective tissue disorders is the Ehlers-Danlos syndrome type VII that results from mutations in the substrate collagen type 1 or in its type 1 procollagen N-proteinase (type VIIC). In both cases the N-terminus of the procollagen type 1 is not removed and the mutant collagen is deposited into the extracellular matrix with severe dermal and internal organ consequences [29,30]. In ADCL it was shown that the altered and extended C-terminus resulted in increased endoplasmatic reticulum stress (due to protein misfolding), increased coacervation and elastin globule formation leading to lower amounts of insoluble, mature elastin. Enhanced TGFβ signaling is probably secondary to altered elastic fiber anatomy [3].

Two reports indicated molecular heterogeneity in ADCL. In a single ADCL patient, a tandem duplication in the *FBLN5* gene was shown to result in secretion of mutant protein, suggesting a dominant negative effect [11]. However, mutant protein integration in the extracellular matrix has been shown in *FBLN5* related ARCL type 1 [31] and carriers of these mutations do not have a cutis laxa phenotype. A second report showed a mutation in exon 25 of the *ELN* gene which encodes a conserved cross-linking domain important for insoluble elastin formation [10]. This patient also presented with severe neurological involvement, which was not attributed to his cutis laxa syndrome. His father carried the same alteration, but did not exhibit any phenotype. Future confirmation of these two defects may shed further insights on the molecular heterogeneity of ADCL. Until then, ADCL can be considered as a phenotypically and genetically homogeneous disorder in which analysis of exon 30-34 of the *ELN* gene is indicated in first instance.

## Conclusion

This cohort of patients further indicates that ADCL is a systemic disorder with a risk for major complications including aortic root dilatation and pulmonary emphysema, warranting regular follow-up. The molecular data favor a single mutational mechanism resulting in a stable C-terminal extension of the elastin protein.

## Abbreviations

ADCL: Autosomal dominant Cutis Laxa; ARCL: Autosomal recessive cutis laxa; ARD: Aortic rooth dilatation; ELN: Elastin; SVAS: Supravalvular aortic stenosis; WBS: Williams-Beuren syndrome; TGFβ: Transforming Growth Factor β

## Competing interests

The authors declare no competing interests.

## Authors’ contributions

SHR and BLC reviewed patients, designed the study and drafted the manuscript. EB, MK, ASP, VL and DB reviewed patients. MR and PJC performed and supervised the molecular analyses. JDB, FM, OMV and ADP contributed to clinical assessment, sample collection and drafting of the manuscript. CB reviewed patients, and contributed in the design of the study. All authors have read and approved the final manuscript.
